# Comparison of two digital PCR platforms for quantification of genetically modified soybean events

**DOI:** 10.1080/21645698.2025.2583781

**Published:** 2025-11-12

**Authors:** Daniela Verginelli, Sara Ciuffa, Katia Spinella, Davide La Rocca, Daniela Villa, Alessandra Barbante, Elena Perri, Ugo Marchesi

**Affiliations:** a National Reference Laboratory for GM Food and Feed, GMO Unit, Istituto Zooprofilattico Sperimentale del Lazio e della Toscana “Mariano Aleandri”, Rome, Italy; b Council for Agricultural Research and Economics, Research Centre for Plant Protection and Certification – CREA DC, Sede di Tavazzan Tavazzano, Italy

**Keywords:** Digital PCR, GMO detection, soybean, validation

## Abstract

In the European Union, the food and feed containing more than 0.9% of approved genetically modified organisms (GMOs) per ingredient must be labeled before placed on the market. In this legislative context, the official control laboratories have to perform validated PCR assays, according to the principles and requirements of ISO/IEC 17025 standard, regarding event-specific methods for the detection, identification and quantification of GMOs. In recent years, with the advent of digital PCR (dPCR) techniques, a growing number of laboratories have transferred the previously validated real-time PCR testings into a dPCR format. Compared to real-time PCR, the dPCR offers the advantage to provide accurate quantification without the need for external calibration samples, show less sensitivity to PCR inhibitors and is more suitable for multiplexing. In this study, an *in-house* validation of quantitative duplex dPCR methods was performed involving MON-04032–6 and MON89788 assays with the lectin reference gene, on the two different platforms Bio-Rad QX200 and Qiagen QIAcuity. All evaluated data and the validation parameters agree with the acceptance criteria validation performance parameters according to the JRC Guidance documents and technical reports in both platforms. The duplex PCR methods here investigated are equivalent in terms of performance to the singleplex real-time PCR method and suitable to perform a collaborative trial for a full validation.

## Introduction

Since the first introduction of GM crops in 1996, their production has shown a continuous and steady increase; as of 2023, the global area of GM crops had reach 206.3 million hectares, with 27 collectively cultivating 11 different GM crops. Among the GM crops, soybeans are the most widely planted at 100.9 million hectares^1^. In Europe, the presence of genetically modified organisms (GMOs) in food and feed products is regulated, imposing a threshold for labelling of products containing more than 0.9% of authorized GMOs per ingredient (Reg. CE 1829/2003 and Reg. CE 1830/2003)^2,3^. According to the Reg. CE 1829/2003,^2^ the developer of a GMO must provide a method for quantifying specific GM events and fund a collaborative trial to validate the method. Currently, all GMO analytical methods submitted for validation to the European Union Reference Laboratory for Genetically Modified Food and Feed (EURL-GMFF) (https://gmo-crl.jrc.ec.europa.eu/) have been developed and optimized for real-time PCR (qPCR). Validated qPCR methods are widely used in European control laboratories, and they must act according to the requirements of the ISO/IEC 17025 standard^4^.

However, qPCR is prone to the presence of inhibitors in DNA templates, and standard curves based on simplex qPCR analyses are no longer sufficiently cost-effective as the number of GMOs increased over time^5,6^. Digital PCR (dPCR) is a sensitive nucleic acid quantification PCR technology that works by portioning DNA into tens of thousands of separate reactions called partitions, and then measuring the endpoint fluorescence of each partition to determine the presence (1) or absence (0) of the target^7,8^. This makes dPCR less reliant on the kinetics of the PCR and eliminates the need for standard curves, similar to qPCR. Statistical methods (Poisson distribution) have been used to calculate the absolute concentration of the target based on the number of positive and negative partitions^8^. Different sample-partitioning models are available based on several approaches, including microfluidic chips, oil-water emulsions, spinning microfluidic discs, microarrays, and microfluidic nanoplates.^9–11^

Using droplet digital PCR (ddPCR) (e.g., Bio-Rad QX200 ddPCR system),^12^ droplets were obtained in a water – oil emulsion to form partitions that separated the template DNA molecules. All reagents were dispersed in nanoliter-sized droplets, and each droplet represented a PCR. After the end-point PCR, each droplet was individually analyzed using an optical detection system. In nanoplate dPCR (e.g., Qiagen QIAcuity One system),^13^ partitioning was achieved using a dedicated plate. The dPCR system integrates partitioning, thermocycling, and imaging into a single dPCR instrument^14^. Digital PCR data analysis was performed using software that calculates the results in copies of the target sequence per microliter of reaction. Overall, this method is currently employed in various analytical fields where the quantification of nucleic acid targets is required and is widely adopted in GMO detection and quantification.^15–18^ Several scientific papers,^19–24^ guidelines,^8, 25–27^ and international standards^26^ provide the requirements for the evaluation of the performance of quantification methods by qPCR and dPCR, and verification by digital PCR has been published.

Previous studies have shown the feasibility of the direct transfer of qPCR validated methods by the EURL-GMFF (European Reference Laboratory for GM Food & Feed, http://gmocrl.jrc.ec.europa.eu/gmomethods/.) for analysis of GMOs, to a dPCR system.^21,22^

This study aimed to compare two different dPCR platforms to carry out an *in-house* validation to detect two of the most abundant GM soybean lines on the world market, the transgenic event known commercially as the “Roundup Ready” and MON89788 GM soybean (Unique identifier MON-Ø4Ø32–6 and MON-89788–1). However, for the MON89788 ddPCR duplex assay, *in-house* validation on the Bio-Rad platform has already been published.^21^ These qPCR methods have been validated by the EURL-GMFF.^28,29^ and are routinely used for official control by national reference laboratories (NRLs). To perform a direct comparison between Bio-Rad’s QX200™ Droplet Digital™ PCR System and Qiagen’s QIAcuity digital PCR system for each method, an identical primer-probe set was used. Optimization of the reaction conditions and method verification were performed according to published guidelines.^8, 25–27, 30^ dPCR performance was assessed in the context of specificity, cross-talk, robustness, dynamic range, linearity, asymmetric limit of quantification (LOQ_asym_), and accuracy (trueness and precision). Moreover, the measurement uncertainty (MU) was evaluated as reported in the ENGL’s document.^31,32^

## Material and Methods

### DNA Extraction and Assessment of DNA Purity

The MON-04032–6 SOYA BEAN ERM-BF410bp (> 985 g/kg), ERM-BF410cp (1 g/kg), ERM-BF410dp (10 g/kg), ERM-BF410ep (100 g/kg), and non-modified soybean ERM-BF410ap (< 0.09 g/kg) CRMs were purchased from the Joint Research Centre (JRC) of the European Commission (Geel, Belgium), while MON89788 soybean (AOCS 0906-B2 ≥996 g/kg) and non-modified soybean (AOCS 0906-A <0.8 g/kg) were purchased from the American Oil Chemists’ Society (AOCS, Urbana, IL, USA). DNA was extracted from 200 mg of CRMs using the RSC PureFood GMO kit with the extractor Maxwell®RSC Instrument (Promega Madison, WI, USA), according to the manufacturer’s instructions for the Bio-Rad platform and using a DNA extraction method with 2% CTAB buffer as described in ISO21571:2005 for the Qiagen platform. DNA concentration was measured by dPCR to evaluate the copy number of the endogenous reference gene lectin (*lec*) using an inhibition test. The inhibition test was carried out at three serial dilution levels, with each level measured in duplicate.^33^ The average of the absolute copies per reaction measured in the diluted samples multiplied by the dilution factor did not differ more than 25% from the average of the absolute copies per reaction measured at the highest concentration (lowest dilution)^33^. Dilutions of extracted stock DNA solutions were prepared in nuclease-free water (Sigma-Aldrich Chemie GmbH, Munich, Germany). All DNA extracts were stored at +4°C until subsequent use.

### Sample Preparation

The validation study involves testing several GM levels (% m/m) for MON-04032–6 and MON89788, those not available on the market as certified reference materials were produced by mixing the positive material (GM) with non-GM material.

The materials with 2%, 0.5%, and 0.05% levels (m/m) of GM MON-04032–6 were produced by dilution, respectively, of the 10%, 1% and 0.1% GM (m/m) with pure non-GM material. Similarly, the GM levels (10%, 2%, 1%, 0.5% and 0.1% GM (m/m)) of MON89788 were determined as described by Hougs *et al*., 2017 (annex 3).^33^ Mixtures were prepared by considering the absolute copy number of the *lec* reference gene measured by dPCR.

### Workflow and Data Analysis of dPCR Analysis

**Figure 1.**
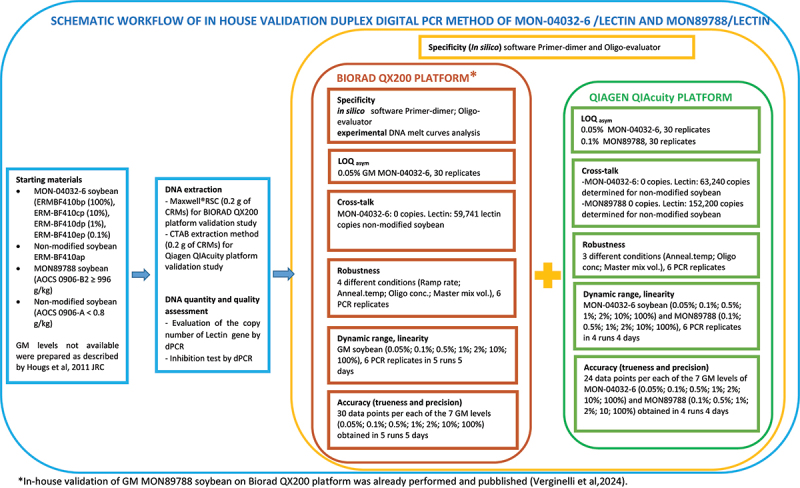
Schematic workflow representing the experimental design of the study.

The *in-house* validation of GM soybean MON89788 and MON-04032–6 was performed on two different digital PCR platforms: QIAcuity dPCR (QIAGEN) and QX200 Bio-Rad. The MON89788 ddPCR assay was previously validated by Verginelli *et al*.,^21^ and then the optimized assay was then transferred to the QIAcuity platform (Figure 1).

QIAcuity dPCR (QIAGEN) was performed on a microfluidic QIAcuity Nanoplate (QIAGEN) using a fully integrated system with partitioning, thermocycling, and imaging performed on a single instrument. For qPCR, reaction mixtures were prepared and loaded onto Nanoplate 26k which provides 24 reactions with 26,000 partitions per well. After sealing, the nanoplate was loaded onto the QIAcuity One, which features a five-channel optical format. Thermocycling was performed, the partitions for each well were imaged, and data analysis was performed using the QIAcuity Software Suite (QIAGEN). On the contrary, using the QX200™ ddPCR Systems (Bio-Rad Laboratories), partitions were generated via water–oil emulsion with a droplet generation cartridge and the droplets were then transferred onto a traditional 96-well plate format. This workflow requires the preparation of each reaction well, similar to qPCR, followed by transfer of each reaction into a droplet generation cartridge. Droplets were generated using a QX200 Droplet Generator (Bio-Rad Laboratories, Inc, Berkeley, CA, USA). After the thermocycling phase, the plate was transferred to a QX200 Droplet Reader, and the droplets for each reaction well were read. A quality assessment of each partition and reading of the endpoint fluorescent signal in two available channels for partitions meeting the required quality parameters were included. The data produced were analyzed and managed using QX Manager™ 2.1 Software (Bio-Rad Laboratories).

### Optimization of the dPCR Conditions

The PCR was conducted based on officially validated qPCR protocols for MON-04032–6^34^ and MON89788^35^ quantification, with some modifications. To quantify MON-04032–6 in 2011, the Italian National Reference Laboratory (NRL) organized an interlaboratory study within the Italian network, including 17 laboratories.^36^ The EURL-GMFF published method records a temperature of annealing and extension at 60°C in both the taxon-specific and the event-specific systems, whereas original EURL-GMFF method reported different annealing temperatures between GM and reference gene systems (60°C for lectin and 55°C for MON-04032–6 soybean). Initially, to transfer the qPCR method to the ddPCR QX200 Bio-Rad, the annealing and extension temperature was tested at 60°C. But, due to an unclear cluster separation between positive signals and background noise, an annealing temperature of 55°C was adopted. The primer and/or probe concentrations were then optimized. The final concentrations of primers and probes are listed in Table S1. Figure S1 shows the optimization of the annealing and extension temperatures, along with the primer and probe concentrations, for the MON-04032–6/lectin and MON89788/lectin.

### QX200 ddPCR System

PCR was performed using a QX200 ddPCR apparatus (Bio-Rad, Pleasanton, CA, USA). The reaction volume was 20 μl containing 1× ddPCR Supermix for probes (No dUTP) (Bio-Rad, Pleasanton, CA, USA), 4 μl of DNA, 600 nM of primers, and 250 nM of probes, for both the Lec and MON-04032–6/MON89788 SOYA BEAN assay (Table S1). Droplets were generated in DG8 cartridges (Bio-Rad) loaded onto a QX200 Droplet Generator (Bio-Rad Laboratories, Inc.). Droplets were then transferred to 96-well plates amplified in a PCR thermocycler Veriti™ Thermal Cycler, 96-well Fast (Thermo Fisher Scientific, Waltham, MA, USA) with the following thermal profile: 10 min DNA polymerase activation at 95°C, 45 cycles of a two-step thermal profile of 30 s at 95°C for denaturation, and 60 s at 55°C for annealing and extension, droplets stabilization at 98°C for 10 min followed by an infinite hold at 4°C. After thermal cycling, the 96-well plates were transferred to a QX200 Droplet Reader (Bio-Rad Laboratories, Inc.) and data were analyzed using the QX Manager 2.1 Standard Edition (Bio-Rad Laboratories, Inc.). The results obtained fulfill the following acceptability criteria^8^ to be included in the subsequent elaborations: single peak of amplitude for the positive droplets, a number of accepted droplets above 10.000 per well per 20 μl reaction with clear discrimination between positive and negative signals, the negative control (NTC) with ≤2 fluorescent droplets and the positive control and sample were >2 fluorescent droplets. The final concentration of the target was expressed in unit copies of the target sequence per microliter of reaction.

### QIAcuity dPCR System

MON-04032–6 and MON89788 were quantified using a QIAcuity One dPCR System (Qiagen). The reactions were conducted using 4x Probe PCR Master Mix, template DNA, and primers and probes at the same concentration as those used with the QX200 platform.

The reaction mixture with the DNA template was loaded into the QIAcuity Nanoplate 26k (24-well) (Qiagen, Hilden, Germany) in a total volume of 40 µl. The plate was then closed using the seal provided in the QIAcuity Nanoplate Kit (Qiagen, Hilden, Germany). The thermal cycling conditions were as follows: for the MON-04032–6 soybean assay (I) PCR initial heat activation at 95°C for 2 min, (II) 40 cycles of denaturation at 95°C for 15 s and annealing at 55°C for 30 s; for the MON89788 assay (I) PCR initial heat activation at 95°C for 2 min, (II) 55 cycles of denaturation at 95°C for 15 s and annealing at 60°C for 30 s. To check the absence of cross contamination during the plate assembly, a no-template control (NTC) was amplified in each PCR run. QIAcuity Software Suite 2.5.0.1 (Qiagen, Hilden, Germany) was used to analyze fluorescence data. The images were exposed for 500 ms with a gain of 6. The final concentration of the target was expressed as copies of the target sequence per microliter of the reaction.

The QIAcuity Software Suite reports the concentration of the target (copies/μl) with a confidence value at 95% confidence interval^13^.

### Determination of dPCR Performance Parameters

The *in-house* validation study was evaluated according to the requirements defined in the document “Definition of Minimum Performance Requirements for Analytical Methods of GMO Testing Part 2 European Network of GMO Laboratories European Union Reference Laboratory for Genetically Modified Food and Feed – Part 2”^25^. The critical parameters assessed were specificity, cross-talk, robustness, dynamic range, linearity, asymmetric limit of quantification (LOQ_asym_), accuracy (trueness and precision), and measurement uncertainty (MU). Six replicates per GM level tested for MON-04032–6 were analyzed by performing five runs over 5 days on the Bio-Rad Q×200 platform. On the QIAcuity platform, six replicates per GM level (% m/m) of MON-04032–6 or MON89788 were tested by five runs over 4 days.

The experimental design and technical approach of each parameter are described in Figure 1. The specificity of the duplex assays was investigated *in silico* using Primer-dimer software (version 2018, http://www.primer-dimer.com.) to predict oligonucleotide hybridization events, and Oligo-evaluator software (http://www.oligoevaluator.com/LoginServlet) to evaluate cross-dimer and hairpin formation.

Specificity was experimentally assessed by carrying out DNA melting curves to verify the presence of the expected pure and single amplicon^37^. The PCR experiment was performed using a Rotor-Gene Q 5plex Platform (Qiagen, GmbH-Hilden, Germany) instrument in a final reaction volume of 20 µl, including the 1X FAST Eva Green qPCR Master Mix (Fisher Molecular Biology, Rome, Italy), 1 ng of genomic DNA, and 300 nM each of reverse and forward primers. Two technical replicates were used for each sample. qPCR amplification was performed with the following program: an initial denaturation of 2 min at 95°C, followed by 40 cycles of 95°C for 5 s, 60°C for 30 s, and 72°C for 20 s, with one fluorescence reading per annealing step. A post-PCR DNA melting curve analysis was carried out using a temperature ramping rate of 0.1°C per step with a 30 s rest at each step.

The robustness of the method was also tested by making slight but deliberate and variable changes to the reaction conditions and assessing their impact on the method performance^25^. For the dPCR method, it is useful to investigate the effects of small changes in reagent concentrations and annealing temperatures, as they can occur due to random pipetting errors and temperature fluctuations in the thermocycler.

Robustness was evaluated at concentration close to the LOQ by applying some modifications to the original protocol. On QX200, four deliberate modifications were considered: (1) a 10% reduction of mastermix volume (18 µl instead of 20 µl); (2) an increase of 1°C of the annealing temperature; (3) the use of a slower ramp rate (+0.5 ºC/s); and (4) a 10% decrease of oligonucleotide concentration.

QIAcuity platform does not allow the ramp rate setting. Therefore, three deliberate modifications were tested: (1) a 10% reduction of mastermix volume; (2) an increase of 1°C of the annealing temperature; (3) a 10% decrease of oligonucleotide concentration.

We also investigated the amount of “rain” (i.e. droplets that have an intermediate fluorescence and do not seem to belong to either the positive or negative population) using the tool described in Lievens *et al*., 2016^38^. The rain was calculated by using “Cloudy-V2-08. R” and an adapted version of “read_QX.R” for output data of QX Manager Software 2.1 Standard Edition. The intermediate signals (rain) should be less than 2.5% of the total number of partitions, as reported by Pecoraro *et al*., 2019^8^.

### Calculation of GM Content

The copy numbers for both event-specific and endogenous reference gene amplifications in each sample were reported using Quanta Soft Software.^12^ Finally, the GMO content in the mass fraction was calculated using the following equation reported in the application note of the European Reference Laboratory (EURL-GMFF).^39^

$$\mathrm{GMcontent}\left(\%\frac{m}{m}\right)=\frac{\left(\mathrm{cp}\;\mathrm{GM}\right)\;}{\left(\mathrm{cp}\;\mathrm{taxon}\;\mathrm{specific}\;\mathrm{sequence}\right)}\times \frac{1}{\left(\mathrm{CF}\;\mathrm{GM}\right)\;}\;\times \;100\;$$

Where *cp GM* is the number of copies of the target per reaction, *cp* taxon-specific sequence is the number of copies of the lectin target per reaction, and *CF GM* is the conversion factor for the certified value of the CRMs used as determined by the EURL GMFF (conversion factors (CFs)) for CRMs.^39^

For QIAcuity the copies per microliter of the target were based on the known number of copies of the target molecule per partition (λ) and the number of accepted partition.

### Measurement Uncertainty (MU)

The measurement uncertainty of the GM content of these materials was determined by considering several uncertainty sources, such as pipetting and the droplet volume in accordance with the guidance for measurement uncertainty for GMO analysis and the European technical guidance document for the laboratories quantifying GMOs operating using a flexible scope of accreditation.^31,32^ The detailed procedure is published by Verginelli et al. 2024.^21^

## Results

### Specificity Evaluation

The specificity of the duplex dPCR assays was verified *in silico* using an analysis software that reported the formation of secondary structures and changes in Gibbs free energy (ΔG) values. A ΔG value smaller than −9 kcal/mol is an absolute indicator of dimer formation. In multiplex PCR, all possible primer combinations can generate unintended PCR products if a matching template is present. No significant risk from dimers was observed between the pairs of oligonucleotides (primers/probes) (Table S2).

Specificity was experimentally evaluated by observing DNA melting peaks in the post-PCR analysis. No additional peaks were observed, thus confirming the absence of unintended products (Figure S2).

### Cross-Talk

The cross-talk test was conducted in the absence of MON-04032–6 and with 59,741 lectin copies determined for non-modified soybean (ERM-BF410ap) in 3 replicates per testing condition using the Bio-Rad QX200 platform. Using the QIAcuity platform, an experiment was conducted using 2275 lectin copies. No cross-talk signals were observed on either platform. As reported in the guidelines for the verification of methods using dPCR,^26^ this performance parameter was considered irrelevant. However, especially with combinations of fluorophore/quencher pairs that are rarely used, depending on the instrument specifications (fluorescence channels), optional testing for optical cross-talk may be helpful.

In the case of MON89778 on the Bio-Rad QX200, this was already verified,^21^ whereas for QIAcuity in the absence of a GM event, 3773 lectin copies were verified.

### Robustness

For both platforms, robustness was evaluated at an LOQ value corresponding to 0.05% for MON-04032–6 and 0.1% for MON89788 (already published for Q×200 in Verginelli *et* *al*., 2024^21^ performing a multifactorial experiment design^40^ as shown in Figure 1.

The small deviations introduced did not significantly affect the measurement results obtained using either platform. The RSDr and bias were estimated to be below 30% at 0.05% and 0.1% GM content, respectively, for the MON-04032–6 and MON89788 soybeans (Table 1).

**Table 1.** Robustness of duplex assay MON-04032–6/Lec (0.05% m/m gm level; 16 conditions) and MON89788/Lec (0.1% m/m; 8 conditions) by dPCR.

Protocol		MON-04032–6						MON89788		
		Biorad QX200			Qiagen QIAcuity			Qiagen QIAcuity		
		Mean (%)	RSDr (%)	Bias (%)	Mean (%)	RSDr (%)	Bias (%)	Mean (%)	RSDr (%)	Bias (%)
Ramp rate change and annealing temp UNCHANGE	Original	0.04	20.10	16.38	0.05	14.85	3.07	0.11	19.18	9.19
	−10% Primer Probe −10% master mix	0.04	22.30	23.10	0.06	5.65	9.56	0.10	25.92	3.97
	−10% Primer Probe	0.04	19.73	29.69	0.05	8.02	0.19	0.12	24.11	24.47
	−10% Master Mix	0.04	20.52	28.91	0.05	8.96	4.55	0.12	22.59	21.51
Annealing temp +1°C	No change PCR reagents	0.04	22.31	18.76	0.05	20.14	1.30	0.09	24.41	5.81
	−10% Primer Probe −10% master mix	0.03	9.31	26.93	0.05	8.80	7.40	0.09	27.13	5.25
	−10% Primer Probe	0.04	8.16	26.05	0.05	17.30	7.40	0.10	19.81	2.93
	−10% Master Mix	0.04	13.71	28.41	0.05	10.40	0.40	0.10	9.80	0.85
Annealing temp +1°C and Ramp rate + 0.5 °C/s	No change PCR reagents	0.04	13.80	26.22	^a^					
	−10% Primer Probe −10% master mix	0.04	11.97	22.45						
	−10% Primer Probe	0.04	9.94	24.69						
	−10% Master Mix	0.04	9.34	29.91						
Ramp rate + 0.5 °C/s	No change PCR reagents	0.04	12.77	29.25						
	−10% Primer Probe −10% master mix	0.04	22.07	18.07						
	−10% Primer Probe	0.04	11.29	19.19						
	−10% Master Mix	0.04	11.81	29.00						

^a^Setting the ramp rate is not available on QIAcuity platform.

### Dynamic Range

The dynamic range was assessed by estimating the trueness and precision, as reported in the ENGL Guidelines.^25,27,30^ The dynamic range for the MON-04032–6 soybean, covering the concentrations from 0.05% to 100% m/m GM level, met the acceptance criteria for precision (RSDr ≤25%) and trueness (Bias ≤25%) on both platforms (Table 2).

**Table 2.** Dynamic range of GM MON-04032–6/Lec and MON89788/Lec was expressed as GM % and the relative values of trueness (Bias %) and precision (RSDr %). The samples were analyzed using duplex dPCR.

	MON-04032–6						MON89788		
	Biorad QX200			Qiagen QIAcuity			Qiagen QIAcuity		
GM level (%)	Measured mean (%)	RSDr (%)	Bias (%)	Measured mean (%)	RSDr (%)	Bias (%)	Measured mean (%)	RSDr (%)	Bias (%)
0.05	0.04	23.38	20.50	0.05	10.79	2.42	/	/	/
0.1	0.08	14.14	21.49	0.09	13.4	14.01	0.11	19.11	9.87
0.5	0.38	9.52	24.35	0.46	23.84	7.89	0.50	18.09	0.08
1	0.77	5.99	22.92	0.81	17.27	18.86	1.10	14.25	10.10
2	1.52	8.91	24.02	1.59	13.61	20.56	2.13	6.26	6.33
10	7.99	1.75	20.06	8.03	5.42	19.66	9.96	3.68	0.38
100	96.14	1.75	2.40	95.34	2.36	4.28	98.67	2.36	0.68

On QIAcuity instrument, the dynamic range for the MON89788 soybean spanning the concentrations from 0.1% to 100% m/m GM level met the acceptance criteria for precision (RSDr ≤25%) and trueness (Bias ≤25%).

### Linearity

Seven levels (% m/m) of MON-04032–6 (0.05%, 0.1%, 0.5%, 1%, 2%, 10%, 100%) and six levels (% m/m) of MON89778 (0.1%, 0.5%, 1%, 2%, 10%, 100%) were measured, and R^2^ and slope between the theoretical and observed values of GM% were evaluated.

R^2^ coefficient should be greater than or equal to 0.98, and the slope should be 1 ± 0.25, as reported in the ENGL guidelines. Duplex assays performed using the Bio-Rad QX200 and QIAgen QIAcuity platforms fulfill the acceptance criteria, exhibiting an R^2^ of 1 and a slope within the acceptable range (Table 3).

**Table 3.** Linearity GM level duplex assays MON-04032–6/Lec (% m/m) and MON89788/Lec (% m/m).

PCR Run	MON-04032-6						MON89788		
	QX200 Biorad			Qiagen QIAcuity			Qiagen QIAcuity		
	Slope	Intercept	R^2^	Slope	Intercept	R^2^	Slope	Intercept	R^2^
R 1	1.02	0.53	1.00	1.04	0.37	1.00	1.02	0.11	1.00
R 2	1.02	0.48	1.00	1.05	0.31	1.00	1.01	0.04	1.00
R 3	1.02	0.48	1.00	1.04	0.38	1.00	1.02	0.08	1.00
R 4	1.02	0.50	1.00	1.03	0.31	1.00	1.00	0.06	1.00
R 5	1.02	0.55	1.00	\	\	\	\	\	\
Mean	1.02	0.51	1.00	1.04	0.34	1.00	1.01	0.07	1.00

**Figure 2.**
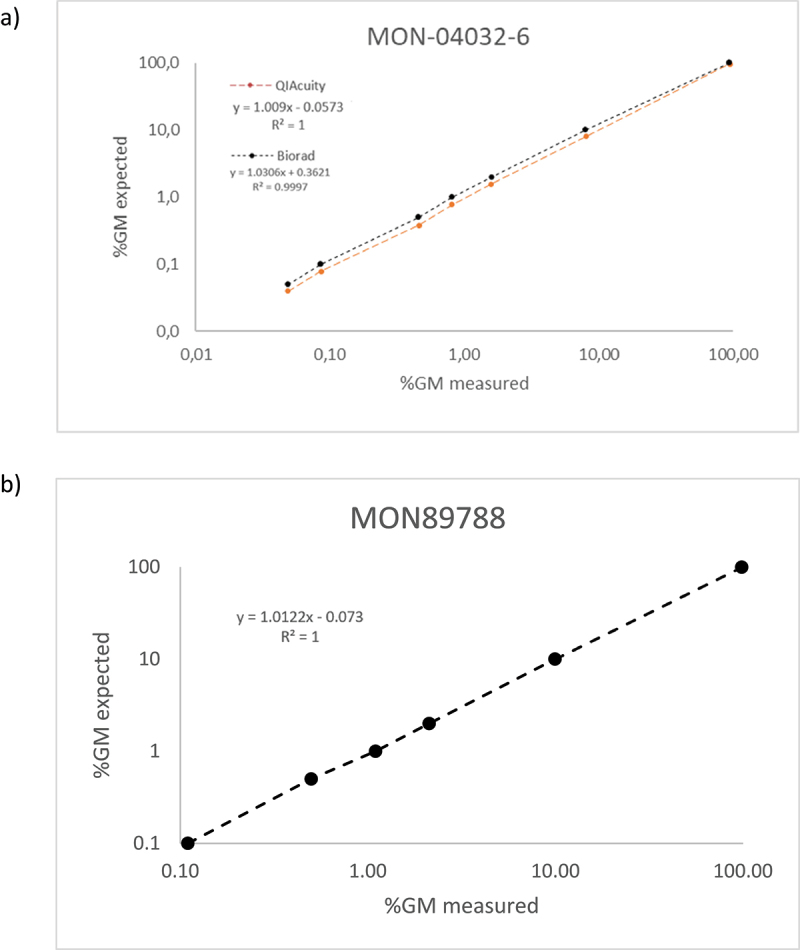
(a) Correlation between measured gm level (% m/m) per reaction of MON-04032–6/Lec using Bio-Rad QX200and Qiagen QIAcuity (b) linearity of gm MON89788/Lec on Qiagen QIAcuity platform.

The correlation between the measured GM levels (% m/m) per reaction for MON-04032–6 and MON89788 is shown in Figure 2.

### Trueness

According to the ENGL guidelines,^25,30^ the acceptance criterion for trueness is that the measured value should be within ±25% of the accepted reference value over the whole dynamic range. The use of certified reference materials is one approach to obtain a suitable reference value, as well as a comparison with results obtained from other methods. The use of another dPCR platform with different MON-04032-6 parameters in greater depth. Trueness, expressed as relative mean bias, was below 25% across the entire dynamic range for the dPCR methods MON-04032–6 and MON89788 for both dPCR platforms, although the value was lower than the QX200 values both cases (Table 4).

**Table 4.** Comparison of performance parameters for the duplex dPCR assays for MON-04032–6/Lec (a) and MON89788/Lec (b) using digital PCR.

a)	Performance parameters																
	MON-04032–6																
	QX200 Biorad								Qiagen QIAcuity								
GM level (%)	N rep	Measured GM (%)	Sr	RSDr%	Ubias	Bias	Bias %	U (%, with k=2)	N rep	Measured GM (%)	Sr		RSDr%	Ubias	Bias	Bias %	U (%, with k=2)
0.05	30	0.04	0.01	23.38	0.04	0.01	20.5	0.004	24	0.05	0.01		10.79	0.01	0.001	2.42	0.001
0.1	30	0.08	0.01	14.14	0.10	0.02	21.49	0.01	24	0.09	0.01		13.40	0.10	0.01	14.01	0.01
0.5	30	0.38	0.04	9.52	0.85	0.12	24.35	0.09	24	0.46	0.11		23.84	0.28	0.04	7.89	0.03
1	30	0.77	0.05	5.99	0.62	0.23	22.92	0.06	24	0.81	0.14		17.27	0.67	0.19	18.86	0.07
2	30	1.52	0.14	8.91	2.22	0.48	24.02	0.23	24	1.59	0.22		13.61	1.15	0.41	20.56	0.12
10	30	7.99	0.14	1.75	5.11	2.00	20.06	0.52	24	8.03	0.44		5.42	5.17	1.97	19.66	0.53
100^a^	30	96.14	1.68	1.75	10.16	2.36	2.40	1.44	24	95.34	2.25		2.36	10.51	4.27	4.28	1.49
b)		MON89788															
		Qiagen QIAcuity															
GM level (%)		N rep	Measured GM (%)			Sr		RSDr%		Ubias		Bias		Bias %		U (%, with k=2)	
0.05		**/**	**/**			**/**		**/**		**/**		**/**		**/**		**/**	
0.1		24	0.11			0.02		19.11		0.32		0.01		9.87		0.04	
0.5		24	0.50			0.09		18.09		0.31		0.00		0.08		0.04	
1		24	1.10			0.16		14.25		2.02		0.10		10.10		0.25	
2		24	2.13			0.13		6.26		0.95		0.13		6.33		0.12	
10		24	9.96			0.37		3.68		2.15		0.04		0.38		0.28	
100		24	98.67			2.33		2.36		5.39		0.67		0.93		0.74	

^a^Corresponding to CRM 99.6% MON-04032–6

### Precision

Precision was estimated by setting up the same experiments that were performed to assess the trueness of the MON-04032–6 and MON89788 dPCR methods. Precision was calculated as RSDr at different concentration levels (% m/m) within the dynamic range under repeatability conditions within the runs and intermediate precision conditions between the runs.

As shown in Table 4, the methods satisfied this requirement for both platforms at all GM levels tested, showing an RSDr spanning from 1.75% on QX200 for the estimation of CRM 100% (m/m) and 23.84% (m/m) for 0.5% (m/m) MON-04032–06 soybean on QIAcuity dPCR.

The RSDr % was higher on QIAcuity platform with the exception of 0.05 GM% (m/m) level of MON-04032–06.

### Asymmetric LOQ (LOQ_asym_)

Thirty replicates at the 0.05% MON-04032–6 were tested for QX200 and for QIAcuity. The determined LOQ_asym_ was 38.96 copies per reaction for MON-04032–6 soya bean (Table S3) on the QX200 Bio-Rad and 25.10 copies per reaction on the QIAcuity Qiagen. For MON89788 in the QIAcuity assay, the LOQ _asym_ value was 16.6 copies per reaction. These results were compliant with the minimum performance parameters, showing an acceptable level of precision expressed in terms of RSDr (≤25%) for both digital PCR platforms (Table S3).

## Discussion

The growing number of GM events on the market requires rapid, fast and efficient methods for detection and quantification of GM them. Among numerous authorized soybean GM events, MON-04032–6 and MON89788 are the most common and specific quantification methods already validated by JRC^34,35^ using real-time PCR approach. In this study, we aimed to compare two digital PCR platforms: the nanoplate‐based Qiagen QIAcuity Digital PCR and the Bio-Rad QX200 Droplet Digital PCR^12,13^.

In this work, the qPCR assays were optimized and transferred in duplex digital PCR methods; moreover, the transferability was also evaluated on two dPCR platforms: QX200 Bio-Rad and QIAcuity Qiagen.^12,13^ Both platforms allow the absolute quantification of the target DNA template without the need for calibrators and standard curves, thus overcoming the shortcomings of quantitative PCR (qPCR). In digital PCR, the target quantification is obtained by dividing a bulk qPCR-like reaction mixture into numerous plenty of individual reactions called partitions; each partition is subjected to an end point PCR reaction. The number of positive partitions is directly related to concentration, and the absolute quantification is determined using the Poisson statistics. The partitioning approach is the main difference between the dPCR platforms used in this study. Actually, the absolute quantification of the target on the Bio-Rad apparatus is based on water-emulsion droplet, while on the QIAcuity dPCR system, the absolute quantification is obtained by microfluidic nanoplate technology. This different approach generates two workflows, with different technical features; e.g. samples/plate, partitions/well and input volume. For this reason, the need to compare the performance of the two platforms was identified and evaluated with this study, showing a good transferability of the MON-04032–6 and MON89788 GM soybean assays, with minor and mandatory modifications (e.g. reaction volume, mastermix). This study represents the first assessment of the GMO quantification on the QX200 and QIAcuity systems, two dPCR platforms with different fluidic partition systems and different histories; the Bio-Rad system was one of the first dPCR systems placed on the market, while QIAcuity was introduced later with a different usage approach. Fluidic partitioning is different between the two apparatuses; ddPCR has a droplet-generating system and a counter to evaluate and count the generated droplets, whereas QIAcuity has physical support for partitioning. In ddPCR, the system is optimized for at least 20,000 droplets of proper size, whereas the Qiagen apparatus uses plates with a physical partition system and allows different number of partitions to be used. The manufacturer recommends a 24,000 partitions plate for low-target quantification and GM testing. These different fluidic systems varied in terms of their outcomes. In this study, the difference in results was validated with respect to the ENGL guideline parameters, showing acceptable validation results.

Demeke *et* *al*., 2025^18^ reported the factors that must be considered for multiplex digital PCR detection: DNA quality, quantity, presence of inhibitors, and droplet or partition volume. These elements can affect the accuracy and uncertainty of dPCR measurements^41^. The Qiagen dPCR system does not allow the setting of temperature gradients, which may lead to potentially inaccurate annealing during the optimization phase. Overall, we observed no annealing issues. Moreover, the robustness study conducted without setting the ramp rate confirms the validity of the results of both platforms. Overall, together with the evaluation of the “rain” we conclusively state that no inaccurate annealings were observed during the dPCR reactions.

The same CRMs were used for this validation study, and DNA was extracted using two different techniques. DNA quality was verified by performing an inhibition test to evaluate the copy number of the lectin reference gene, as described in the Materials and Methods. The observed differences were extremely small, indicating that the DNA extraction step may have a negligible impact, especially when simple matrix materials such as CRMs are analyzed.

All validation parameters, including specificity, cross-talk, robustness, dynamic range, linearity, asymmetric limit of quantification (LOQ_asym_), accuracy (trueness and precision), and measurement uncertainty (MU), on droplet digital PCR and digital PCR in nanoplates have demonstrated comparable results in compliance with international standards^42,43^ and are in agreement with the ENGL document.^8,25,27^ The dPCR methods on the two platforms were suitable for the application of both dPCR systems for the detection and quantification of GM events for regulatory compliance.

In conclusion, digital PCR quantification of MON-04032–6 and MON89788 in a duplex assay with respect to the endogenous reference gene lectin showed concordance in terms of validation parameters between the QX200 and QIAcuity systems. Overall, this outcome highlights the suitability of the two platforms for full validation by official control laboratories.

## Supplementary Material

Supplemental Material

## Data Availability

Data associated with this manuscript can be accessed online at …
