# Supplementary material for: Comparison of two digital PCR platforms for quantification of genetically modified soybean events
Source: GM Crops Food. 2025 Nov 12;16(1):890–902. doi: 10.1080/21645698.2025.2583781 (PMC12622311; doi:10.1080/21645698.2025.2583781)
Supplement: Supplemental Material [file KGMC_A_2583781_SM2149.docx]

Tab. S1. Specifications of primer and probe system.

| **Target** | **Primers and probes** | **Sequences** | **Final concentration (pmol/μl)** | | **References** |
| --- | --- | --- | --- | --- | --- |
|  |  |  | **Real time PCR** | **QX200 and QIAcuity dPCR** |  |
| MON-04032-6 (84 bp) | 40-3-2 AF | 5’- TTC ATT CAA AAT AAG ATC ATA CAT ACA ggT T- 3’ | 0.3 | 0.6 | QT-EVE-GM-005 (https://gmo-crl.jrc.ec.europa.eu/gmomethods/show/QT-EVE-GM-005/) Gatto et al., 2011 https://www.izslt.it/crogm/wpcontent/uploads/sites/5/2019/04/CROGM_report_screening-1.pdf |
|  | 40-3-2 AR | 5’ggC ATT TgT Agg AgC CAC CTT–3’ | 0.3 | 0.6 |  |
|  | 40-3-2 AP | 6-FAM 5'-CCT TTT CCA TTT ggg - 3’ NFQ-MGB | 0.1 | 0.25 |  |
| MON89788 (139 bp) | MON 89788-F | 5’- TCC CgC TCT AgC gCT TCA AT- 3’ | 0.15 | 0.6 | QT-EVE-GM-006 (https://gmo-crl.jrc.ec.europa.eu/gmomethods/show/QT-EVE-GM-006/) Verginelli, D. et al., 2024. https://doi.org/10.3390/foods13244011 |
|  | MON 89788-R | 5’- TCg AgC Agg ACC TgC AgA A- 3’ | 0.15 | 0.6 |  |
|  | MON 89788-P | 6-FAM 5'-CTg AAg gCg ggA AAC gAC AAT CTg- 3’ BHQ1 | 0.05 | 0.25 |  |
| Lec (74 bp) | Lec-F | 5’- CCA gCT TCg CCg CTT CCT TC –3’ | 0.3 | 0.6 | QT-TAX-GM-002 (https://gmo-crl.jrc.ec.europa.eu/gmomethods/show/QT-TAX-GM-002/) |
|  | Lec-R | 5’- gAA ggC AAg CCC ATC TgC AAg CC –3’ | 0.3 | 0.6 |  |
|  | Lec-P | HEX 5’- CTT CAC CTT CTA TgC CCC TgA CAC 3’BHQ1 | 0.1 | 0.25 |  |

a)


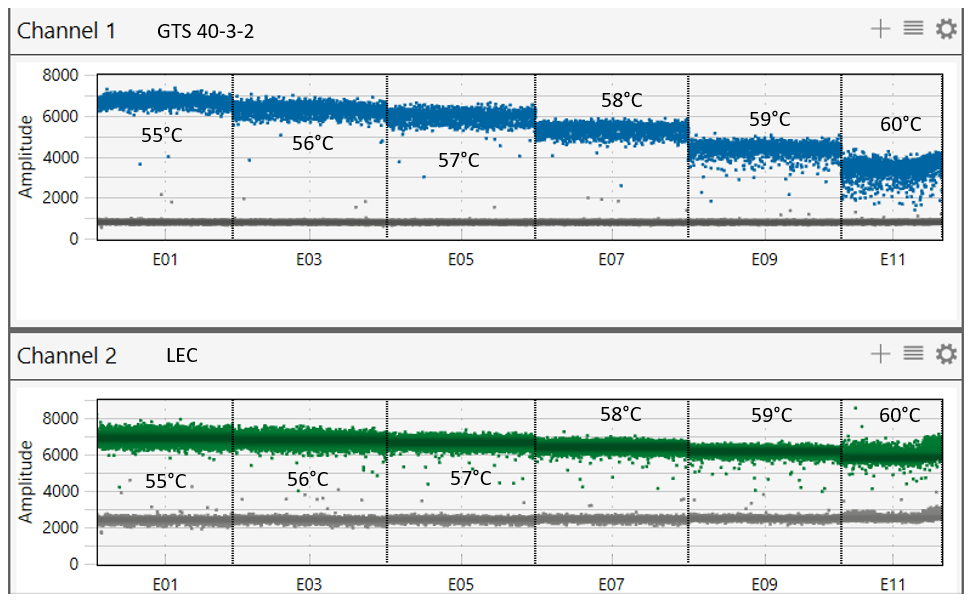


LECTIN

MON-04032-6

b)


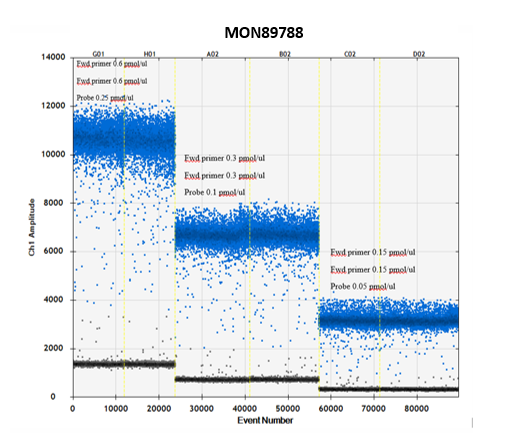


Fig. S1. QX200 output. Optimization of annealing temperature and primer/probe concentration in the duplex assays of MON-04032-6/lectin (a) and MON89788/lectin (b)

Tab. S2. Specificity evaluation of MON-04032-6 /Lec methodᵃ. a) Outcome of self-dimer evaluation using Primer-dimer software; b) Outcome of cross-dimer evaluation using Oligo-evaluator software.

a)

| **Forward Primer Name** | **Forward Primer Seq** | **Reverse Primer Name** | **Reverse Primer Seq** | **Structure** | **ΔG** |
| --- | --- | --- | --- | --- | --- |
| 40-3-2AF | TTCATTCAAAATAAGATCATACATACAGGTT | 40-3-2AR | GGCATTTGTAGGAGCCACCTT | heterodimer | -1.98 |
| 40-3-2AF | TTCATTCAAAATAAGATCATACATACAGGTT | 40-3-2AP | CCTTTTCCATTTGGG | heterodimer | -0.36 |
| 40-3-2AR | GGCATTTGTAGGAGCCACCTT | 40-3-2AP | CCTTTTCCATTTGGG | heterodimer | -0.36 |
| LecF | CCAGCTTCGCCGCTTCCTTC | LecP | CTTCACCTTCTATGCCCCTGACAC | heterodimer | 0 |
| LecR | GAAGGCAAGCCCATCTGCAAGCC | LecP | CTTCACCTTCTATGCCCCTGACAC | heterodimer | -5.28 |
| LecF | CCAGCTTCGCCGCTTCCTTC | LecR | GAAGGCAAGCCCATCTGCAAGCC | heterodimer | -5.28 |
| LecF | CCAGCTTCGCCGCTTCCTTC | 40-3-2AR | GGCATTTGTAGGAGCCACCTT | heterodimer | -1.2 |
| 40-3-2AF | TTCATTCAAAATAAGATCATACATACAGGTT | LecR | GAAGGCAAGCCCATCTGCAAGCC | heterodimer | -5.28 |
| 40-3-2AF | TTCATTCAAAATAAGATCATACATACAGGTT | LecP | CTTCACCTTCTATGCCCCTGACAC | heterodimer | 0 |

b)

| **Name** | **Sequence** | **Base Count** | **Length (bp)** | **Molecular Weight** | **Tm (°C)** | **Extinction Coefficient** | **µg/OD at 260 nm** | **GC%** | **GC Clamp** | **Run Length (bp)** | **Primer Dimer** | **Secondary Structure** |
| --- | --- | --- | --- | --- | --- | --- | --- | --- | --- | --- | --- | --- |
| 40-3-2AF | TTCATTCAAAATAAGATCATACATACAGGTT | A = 13, U = 0, G = 3, C = 5, T = 10, I = 0, Total = 31 | 31 | 9485.3 | 64.2 | 316.7 | 30.0 | 25.8 | 2 | 4 | No | None |
| 40-3-2AR | GGCATTTGTAGGAGCCACCTT | A = 4, U = 0, G = 6, C = 5, T = 6, I = 0, Total = 21 | 21 | 6437.3 | 66.2 | 197.6 | 32.6 | 52.4 | 2 | 3 | No | Moderate |
| 40-3-2AP | CCTTTTCCATTTGGG | A = 1, U = 0, G = 3, C = 4, T = 7, I = 0, Total = 15 | 15 | 4525.0 | 54.2 | 129.3 | 35.0 | 46.7 | 3 | 4 | No | None |
| LecF | CCAGCTTCGCCGCTTCCTTC | A = 1, U = 0, G = 3, C = 10, T = 6, I = 0, Total = 20 | 20 | 5956.0 | 72.3 | 162.1 | 36.7 | 65.0 | 2 | 2 | No | Weak |
| LecR | GAAGGCAAGCCCATCTGCAAGCC | A = 7, U = 0, G = 6, C = 8, T = 2, I = 0, Total = 23 | 23 | 7027.7 | 75.3 | 220.5 | 31.9 | 60.9 | 3 | 3 | No | Moderate |
| LecP | CTTCACCTTCTATGCCCCTGACAC | A = 4, U = 0, G = 2, C = 11, T = 7, I = 0, Total = 24 | 24 | 7159.8 | 68.7 | 206.1 | 34.7 | 54.2 | 1 | 4 | No | Very Weak |

ᵃ The specificity assessment of MON89788 method is reported by Verginelli et al,2024. All of the possible primer/probe combinations were analysed for each duplex, confirming the theoretical specificity of the methods


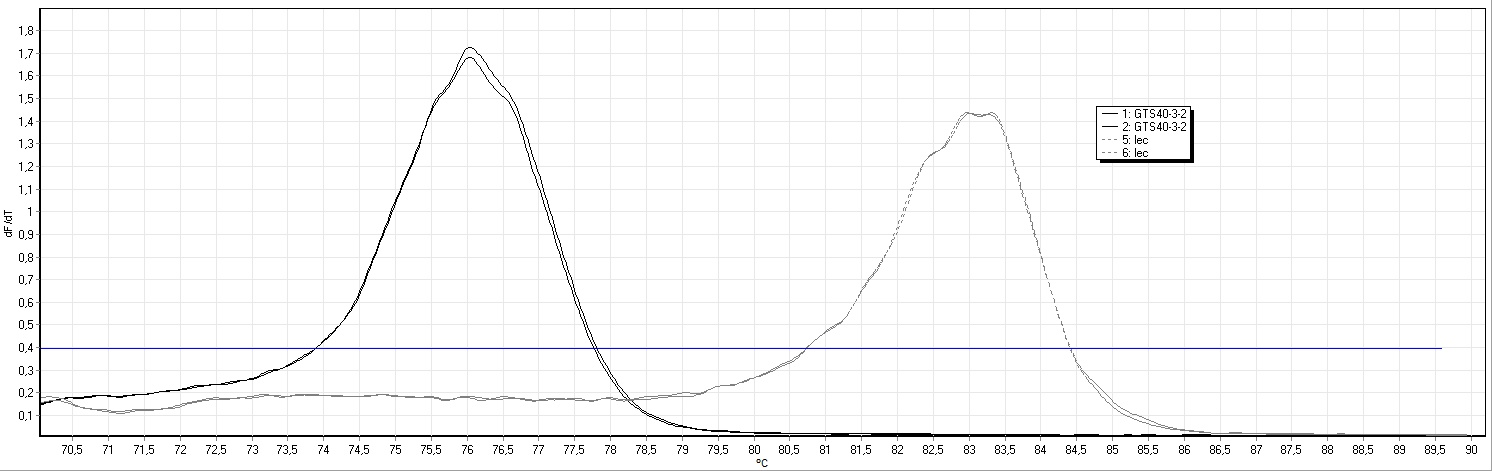


# Fig. S2. Amplicon melting analyses for MON-04032-6 soybean (black line), and lectin soybean endogenous gene lectin (dash line).

# Tab. S3. Determination of LOQasym of the MON-04032-6/Lec and MON89788/Lec using dPCR. Outlier evaluation was determined by Grubb's test.

|  | **MON-04032-6** | | | | **MON89788** | |
| --- | --- | --- | --- | --- | --- | --- |
|  | **Biorad QX200** | | **Qiagen QIAcuity** | | **Qiagen QIAcuity** | |
| Expected GM level (%) | 0.05 | | 0.05 | | 0.1 | |
| Average GM level (%) | 0.04 | | 0.05 | | 0.1 | |
|  | **GM cp/rxn** | **Lectin cp/rxn** | **GM cp/rxn** | **Lectin cp/rxn** | **GM cp/rxn** | **Lectin cp/rnx** |
| Copy number (cp/rxn) | 38.96 | 96607.0 | 25.10 | 50368.0 | 16.6 | 16045.3 |
| RSDr (%) | 23.72 | | 13.50 | | 10.8 | |
| N.outlier | 0 | | 0 | | 0 | |
